# Comparison of Chemical Profiles, Anti-Inflammatory Activity, and UPLC-Q-TOF/MS-Based Metabolomics in Endotoxic Fever Rats between Synthetic Borneol and Natural Borneol

**DOI:** 10.3390/molecules22091446

**Published:** 2017-08-31

**Authors:** Liang Zou, Yan Zhang, Wei Li, Jinming Zhang, Dan Wang, Jia Fu, Ping Wang

**Affiliations:** 1 School of Medicine, Chengdu University, Chengdu 610106, China; zouliang@cdu.edu.cn (L.Z.); zyanTCM@hotmail.com (Y.Z.); liweivirlee@163.com (W.L.); wangdan@cdu.edu.cn (D.W.); 2 Department of Pharmacology, Chengdu University of Traditional Chinese Medicine, Chengdu 611137, China; zhangjinming1987@126.com

**Keywords:** natural borneol, synthetic borneol, GC-MS, anti-inflammatory activity, RAW 264.7 cells, UPLC-Q-TOF/MS, metabonomic

## Abstract

Natural borneol (NB, called “Bingpian”) is an important traditional Chinese medicine to restore consciousness, remove heat and relieve pain, all of which are inflammation-related diseases. Recently, due to the limited source of NB, synthetic borneol (SB) is widely used as a substitute for NB in clinics. However, little is known about the effects of SB instead of NB. Herein, the aim of the present study was to compare NB and SB on chemical profiles by gas chromatography-mass spectrometer (GC-MS) analysis, anti-inflammatory activity in lipopolysaccharide (LPS)-induced RAW 264.7 macrophages, and ultra-performance liquid chromatography-quadrupole time-of-flight mass spectrometry (UPLC-Q-TOF/MS) metabolomic approaches in endotoxic fever induced in rats. Results showed that, in total, 13 volatile components could be identified in NB and SB by GC-MS analysis, in which a significant difference between them still existed. The main constituents in SB were iso-borneol and borneol, while borneol contributes to 98.96% of the amount in NB. Additionally, both NB and SB exhibited remarkable anti-inflammatory effects to reduce the level of inflammatory factors including NO, TNF-α and IL-6 in LPS-induced RAW 264.7 macrophages, and lower the high body temperature in rats with endotoxic fever induced by LPS. Moreover, it seems that NB exhibited higher efficacy than SB. The unequal bioactive efficiency between NB and SB was also indicated by means of non-targeting metabolomics. Based on UPLC-Q-TOF/MS technology, 12 biomarkers in the serum of fever rats were identified. Pathway analysis revealed that the anti-fever effect of NB and SB was related to regulating the abnormal glycerophospholipid, linoleic acid and alpha-linoleic acid metabolism pathways in the fever model. Results indicated that there was still a great difference between NB and SB involving chemical constituents, anti-inflammation activity and the ability to regulate the abnormal metabolism pathways of the fever model. Certainly, further studies are warranted to better understand the replacement rationale in medicinal application.

## 1. Introduction

Natural borneol (NB, Bingpian in Chinese), derived from either *Dryobalanops aromatica Gaertner* or *Cinnamonum camphora* (L.) Presl, is widely applied in traditional Chinese medicine (TCM) as a common and valuable composition in TCM formulae [1]. Several medicinal actions of NB to restore consciousness, remove heat, and relieve pain have been recorded in ancient TCM books, such as the “Compendium of Material Medicine”. It was habitually employed for throat swelling and pain, hot eyes, coma with heat syndrome, chest stuffiness and cardialgia [2]. Likewise, NB is commonly used as an ingredient in many modern Chinese herb therapies to treat cardiovascular, cerebrovascular, ophthalmic and dermal diseases, and even in cosmetics. Recently, a variety of pharmacological activities of borneol has been reported, including neuroprotection [3], sedation [4], and anti-cerebral ischemia [5]. Additionally, borneol could suppress inflammatory responses in lipopolysaccharide (LPS)-induced acute lung injury through inhibition of the NF-κB and MAPKs signaling pathways [6]. Moreover, several studies reported the possible capacity of borneol to enhance the oral bioavailability [7], blood brain barrier permeability [8], and tumor drug resistance reversal of other drugs [9].

However, the plant *Dryobalanops aromatica Gaertner* as the main raw material of NB is largely distributed in Sumatra, Indonesia. The majority of NB in China is imported, and its source is limited. Thus, *Borneolum syntheticum*, synthetically produced from turpentine oil, is widely used in herbal medicine and cosmetics as a substitution for NB. Dextrorotatory borneol is revealed as the main ingredient in NB, in which the proportion of (+)-borneol is over 96%. Greatly unlike NB, *Borneolum syntheticum* (also called synthetic Bingpian, SB) is a mixture of levorotatory borneol ((−)-borneol) and isoborneol [10]. However, a previous study has reported that isoborneol exhibited higher mucosa stimulus and hepatotoxicity than (+)-borneol, which would result in the potential side-effect of SB instead of NB [11]. Moreover, (−)-borneol and (+)-borneol exhibited different effects on the pharmacokinetic profiles of other combined drugs. Luo et al [12] demonstrated that the promotional effect of (−)-borneol on the pharmacokinetic parameters of osthole was greater than that of (+)-borneol. This result indicated that, in view of SB widely being used as an alternative for NB, there would also be some differences between them. 

To evaluate the replacement rationale of SB for NB in the treatment of inflammatory diseases, we compared SB and NB for their chemical constituents and anti-inflammation activity. Based on the volatile compounds in NB and SB, the gas chromatography-mass spectrometer (GC-MS) method was established to investigate the integrated chemical profiles. The anti-inflammation effect was evaluated by LPS-induced RAW 264.7 cells in vitro and LPS-induced rat fever model in vivo. Moreover, metabolomics as a novel “-omics” technology is a scientific method to evaluate and explore the mechanisms of TCM [13]. Coinciding with the integrity and systemic feature of TCM, metabolomics herein was employed to investigate the intervention mechanism of both NB and SB on metabolic profiling of LPS-induced acute inflammation rats. An ultra-performance liquid chromatography-quadrupole time-of-flight mass spectrometry (UPLC-Q-TOF/MS) method was established to determine the change of endogenous metabolites in rat plasma. The comprehensive comparison on chemical and metabolomics profiles would benefit from the evaluation of the substitution rationality of SB for NB.

## 2. Results

### 2.1. Chemical Profiles of SB and NB by GC-MS Analysis

**Figure 1:**
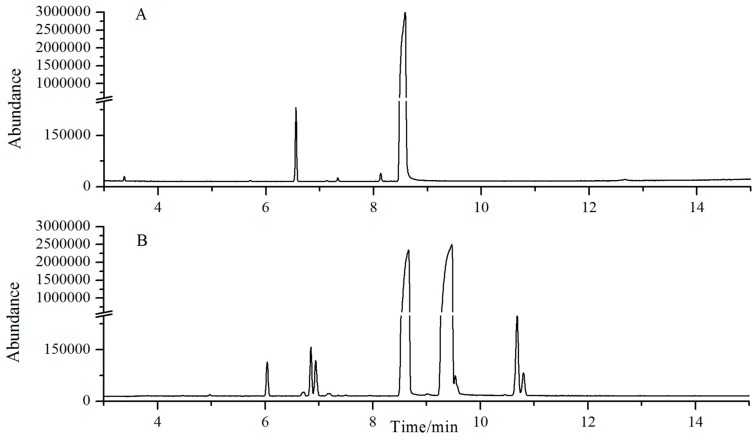
The typical GC-MS analysis chromatogram of NB (**A**) and SB (**B**).

**Figure 2:**
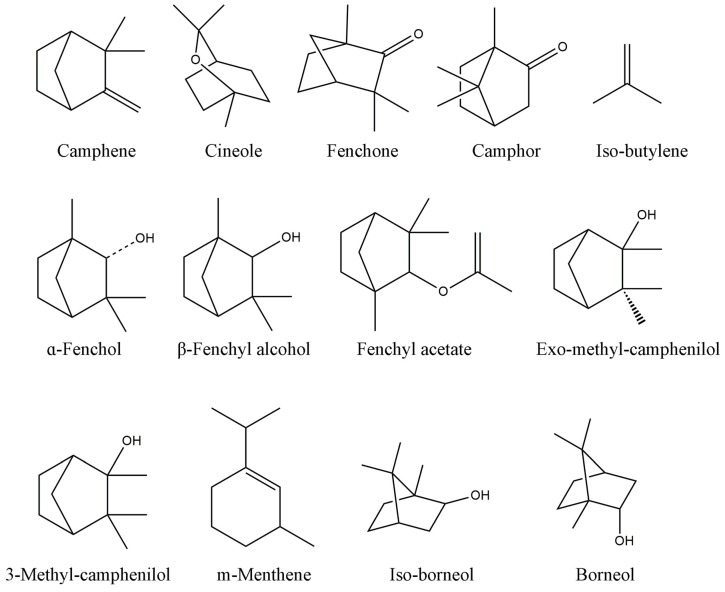
The chemical structure of compounds in NB and SB by GC-MS analysis.

The chemical structure of compounds contained in NB and SB is shown in Table 1 and Figure 1. A typical GC-MS fingerprint of SB and NB is shown in Figure 2. The components of SB and NB are shown in Table 1. Although NB and SB were claimed to have high purity, GC-MS analysis revealed that both NB and SB exhibited a complex chemical profile. A total of fifteen compounds were detected, among which 13 components, including camphene, cineole, fenchone, camphor, iso-butylene, α-fenchol, β-iso-fenchyl alcohol, fenchyl acetate, exo-methyl-camphenilol, 3-methyl-camphenilol, m-menthene, iso-borneol and borneol were identified, accounting for more than 98.0% of the total components of SB and NB. The main compounds in NB were borneol (98.96%). The main compounds in SB were borneol (61.39%) and iso-borneol (35.77%).

### 2.2. Anti-Inflammation Activity In Vitro

**Figure 3:**
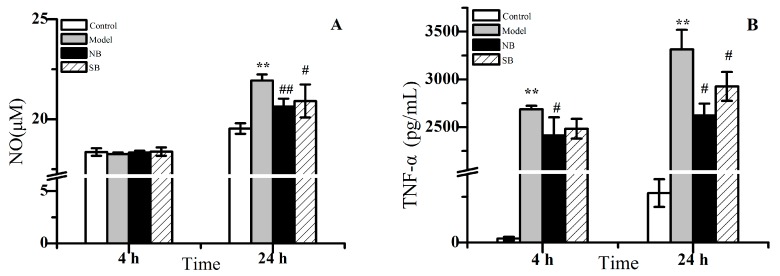

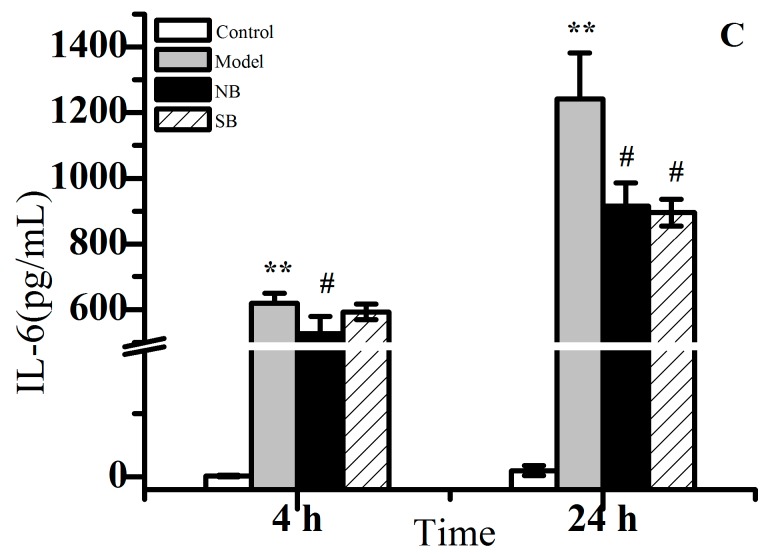
The release of NO (**A**); TNF-α (**B**) and IL-6 (**C**) in lipopolysaccharide (LPS)-induced RAW 246.7 macrophages after being treated by NB and SB for 4 h and 24 h. Note: VS control group, ** *p* < 0.01; vs. model group, # *p* < 0.05 and ## *p* < 0.01.

As shown in Figure 3, the inflammatory cytokines including NO, TNF-α and IL-6 in RAW 264.7 macrophages treated by LPS exhibited a significant increase in a time-dependent manner, compared with the untreated cells (* *p* < 0.05). For example, NO levels in the model group did not display an increase at 4 h of treatment, while a remarkable rise could be found after 24 h of treatment. Additionally, the dramatic rise of both TNF-α and IL-6 in the model group displayed similar behavior. It indicated that the stimulation of LPS for either 4 h or 24 h could definitely result in the inflammation reaction of macrophages. However, after co-treatment with either NB or SB, the inflammation reaction begun to decline in comparison to model cells. Figure 3 provided a clear demonstration that both NB and SB could lead into decreases of NO, TNF-α and IL-6 levels in the LPS-treated RAW 264.7. Although the decreasing tendency could be found after 4 h of treatment, the higher anti-inflammation effect of NB/SB was observed after 24 h of treatment, in which a statistical significance of declines of NO, TNF-α and IL-6 expression was shown (# *p* < 0.05). Nevertheless, there was no remarkable difference between NB and SB, on the decrease effect for inflammatory factor levels. These results indicated that both NB and SB could alleviate the inflammatory reaction induced by LPS in vitro.

### 2.3. Serum Metabonomics of NB and SB in Endotoxic Fever Rats

A standard endotoxic fever rat model has been established by the intravenous injection of the saline suspension of LPS at a dose of 5 mg/kg [14]. Fever started within 2 h after LPS injection. The body temperature of model rats dramatically rises to average 38.2 ± 0.2 °C at 1.5 h post-injection, compared to the initial temperature at an average 37.3 ± 0.2 °C in control rats. Particularly, fever rats orally administrated with either NB or SB displayed a lower body temperature at about 37.8 °C after 1.5 post-injection than the model group. The results of this study demonstrated that both NB and SB could attenuate endotoxic fever in rats, which was in accord with its anti-inflammatory activity in RAW 264.7 macrophages.

**Figure 4:**
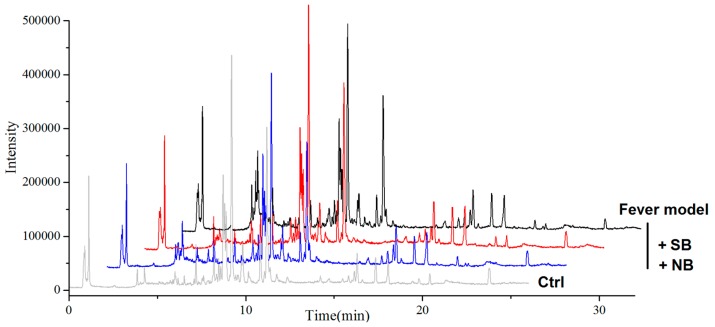
Representative ultra-performance liquid chromatography-quadrupole time-of-flight mass spectrometry (UPLC-Q-TOF/MS) TIC (total ion flow) chromatograms of the samples.

To investigate the potential mechanism related to metabolomic influence by borneol, a non-target serum metabolomics approach was employed to find more metabolite change information, which was closely related to the reliability of results. Primarily, to ensure the reproducibility of the UPLC-Q-TOF/MS analysis method, the same serum sample was analyzed repeatedly, five times for one day and five consecutive days, respectively. These main extracted ions in UPLC-Q-TOF/MS analyses were selected for method validation. By calculating the retention time and *m*/*z* data of the selected ions, the instrument precision, method reproducibility, sample stability and system stability were evaluated. All the relative standard deviation (RSD %) values were less than 5%, showing that the precision, reproducibility and stability of the metabolites analysis method was dependable, and the analysis results were reliable. The serum metabolites profiles were obtained from each rat group in positive ion mode. Representative UPLC-Q-TOF/MS TIC chromatograms are presented in Figure 4.

**Figure 5:**
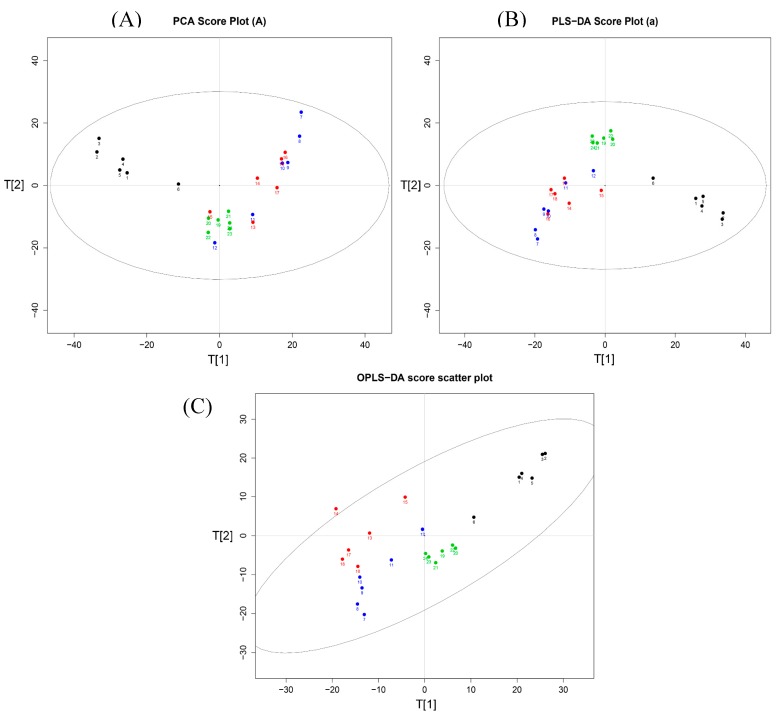
(**A**) Score plots of principle component analysis (PCA), (**B**) partial least-squares discriminant analysis (PLS-DA) and (**C**) orthogonal partial least-squares discriminant analysis (OPLS-DA) analysis on the fever rat serum metabolic profiles of normal control (black dot), model (blue dot), SB treatment (red dot) and NB treatment group (green dot).

**Figure 6:**
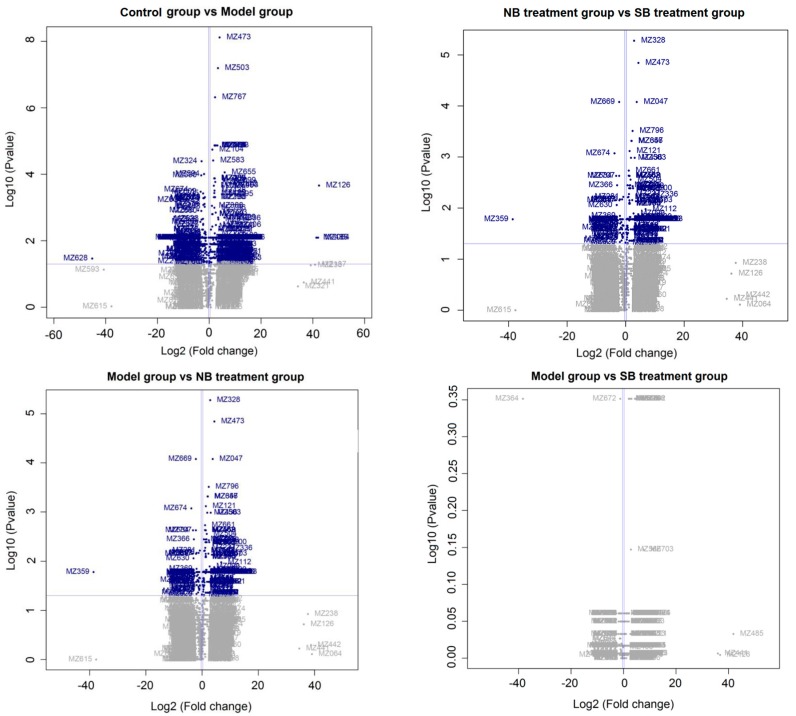
Volcano plot results of identical biomarkers in various groups. Blue variables represent significant results (*p* value < 0.05) and showed fold changes >1.2 or <0.8.

As shown in Figure 5A, the corresponding score plot from principle component analysis (PCA) showed that samples in rats with or without LPS induction were clearly clustered [15]. An absolute separation between untreated control rats and other fever rats in the direction of the first principal component, indicated that the endogenous metabolite profiles of the un-fevered rats were significantly different from that of fever rats. Meanwhile, the serum metabolite profiles from the model group, NB group and SB group also displayed different clusters, suggesting that the administration of either NB or SB could change the metabolites of fever model rats. Although the PCA as an unsupervised pattern recognition method provided an overview of these groups, the variable difference between clusters was still unclear. Subsequently, the supervised pattern recognition approaches [16] like partial least-squares discriminant analysis (PLS-DA) and orthogonal partial least-squares discriminant analysis (OPLS-DA) were used to maximize the discrimination and present the metabolite differences among groups. Figure 5B,C show more clear differences among groups based on the loading and score plots of serum different phases. All profiles in groups, i.e., control group (blank dot), model group (blue dot), NB group (green dot) and SB group (red dot), were clustered well and displayed obvious differences between each other. Nevertheless, the X-variables in the model and SB group were close. Additionally, the volcano plot images in Figure 6 displayed the corresponding results, in which there was a great difference between the control and model groups, and between the NB and model groups, instead of that between the SB and model group.

**Figure 7:**
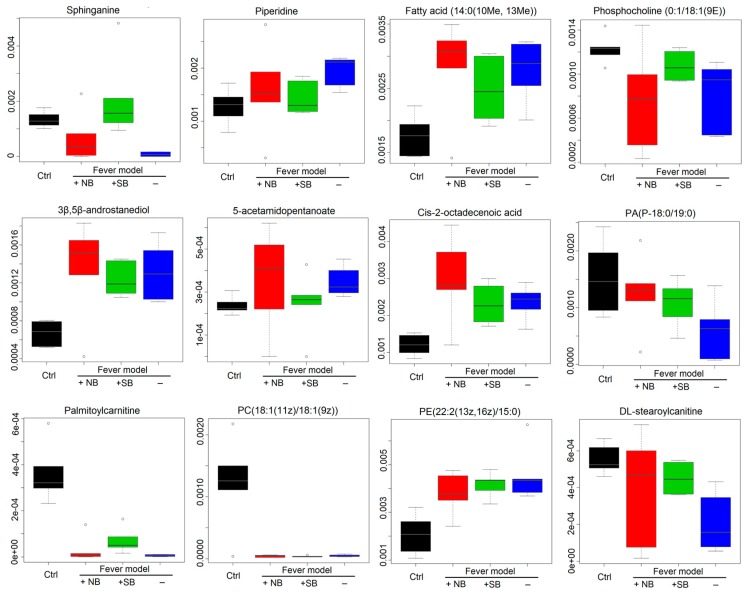
Boxplots of identical biomarkers. The vertical axis represents the chromatography peak intensity of each biomarker in UPLC-Q-TOF/MS analysis normalized to that of the chosen reference peak with the maximum peak area.

**Figure 8:**
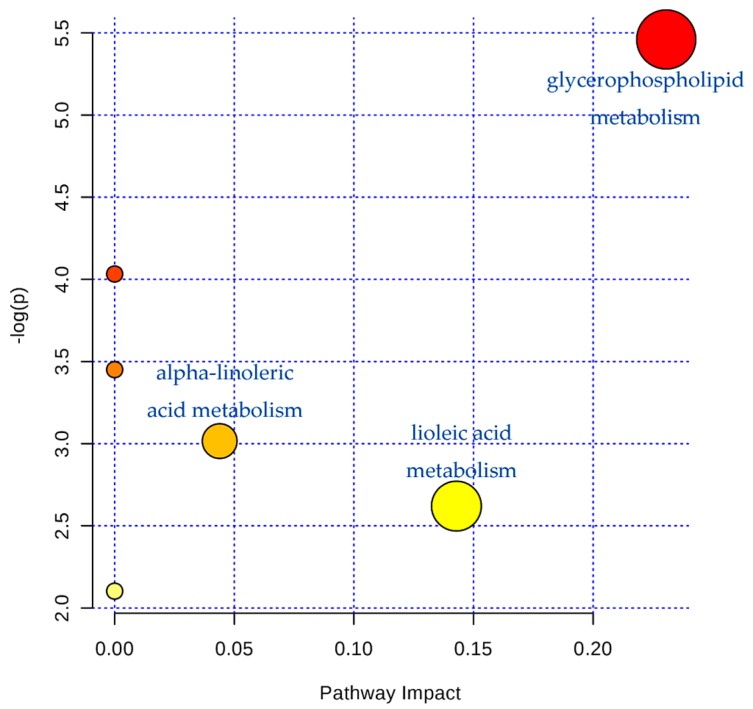
The potential metabolic pathways according to the identified biomarkers by MetaboAnalyst 2.0 analysis. The area of marked circles represents the importance of the metabolic pathway in LPS-induced endotoxic fever rats.

Furthermore, 12 kinds of main endogenous biomarkers were obtained and identified, according to the *m*/*z* result (Table 2), including sphinganine, piperidine, fatty acid(14:0(10Me,13Me), PC (0:1/18:1(9E)), 3β, 5β-androstanediol, 5-acetamidopentanoate, *cis*-2-octadecenoic acid, PA(P-18: 0/19: 0), palmitoylcarnitine, PC(18:1(11Z)/18:1(9Z)), PE (22:2(13Z, 16Z)/15:0), and DL-stearoylcanitine. The different intensity of the various identical biomarkers in serum samples of the various groups are shown in Figure 7. It suggests that the serum metabolic profiles in fever rats were dramatically changed in comparison to normal rats, while NB exhibited a higher efficacy to change the serum metabolic pattern in endotoxic fever rats than SB. Moreover, the potential pathways were detected by MetaboAnalyst 2.0 (Chengdu, China), which indicate that the glycerophospholipid, linoleic acid, and alpha-linoleic acid metabolisms are the main metabolic pathways of LPS-induced fever rats (shown in Figure 8).

## 3. Discussion

Inflammation is a defensive reaction of the organism to irritation, characterized by redness, swelling, heat, and pain. Normally, inflammation is beneficial in tissue repair and is an automatic defense response of the body [17]. However, inflammatory processes may also cause tissue damage, multiple organ failure, and even lead to death [18,19]. Bacterial LPS can cause the activation of the nuclear factor-κB (NF-κB) pathway, which can upregulate pro-inflammatory proteins such as nitric oxide synthase (iNOS) and cyclooxygenase-2 (COX-2) [20]. A large amount of NO was produced by the increasing level of iNOS, and then NO contributes to the synthesis and release of inflammatory factors such as cytokines and derivatives of arachidonic acid, including PGE_2_ [21], and some pro-inflammatory mediators such as TNF-α and IL [22,23]. In general, LPS can cause severe inflammatory reactions in the body and dramatically increase the expression of NO, TNF-α and IL-6 in RAW 264.7 macrophage [24,25,26]. Herein, we evaluated the activity of both NB and SB to inhibit the abnormal rise of these inflammatory factors. As a result, after co-incubation with LPS stimulant for 24 h, both NB and SB exhibited significant anti-inflammation effects (Figure 3). Nevertheless, although there was no remarkable difference between NB and SB on the decrease of inflammatory factor levels, we can still observe that NB results in a relatively lower concentration of NO and TNF-α, in comparison to SB.

The remarkable anti-inflammation efficacy of borneol would result from the chemical constituent in NB and SB. GC-MS analysis data indicated the presence of certain bioactive components of NB, such as borneol (98.96%) and camphor (0.81%), and the main compounds in SB were borneol (61.39%) and iso-borneol (35.77%). Previous studies have affirmed that Abies koreana essential oil (AKE) contains bornyl acetate, limonene, a-pinene, camphene, a-elemene, alloaromadendrene, c-selinene and borneol, and have markedly and dose-dependently inhibitory effects on the activities of LPS-induced NO production by RAW 264.7 cells. AKE reduced the LPS-induced secretion of TNF-α, IL-1β, IL-6, NO and PGE_2_ in RAW 264.7 cells, indicating that it has anti-inflammatory effects [27]. Moreover, bornyl salicylate, the esterification product of salicylic acid and monoterpene (−)-borneol, has been demonstrated to inhibit inflammation at least for 24 hours mainly by reducing pro-inflammatory cytokines [28]. Thyme extracts containing 1, 8 cineole, thymol, camphor, borneol, and carvacrol significantly reduced production and gene expression of the pro-inflammatory factors such as TNF-α, IL-1B, and IL-6 [29]. These data suggest that many substances containing borneol have anti-inflammatory effects, which may be related to the decrease of inflammatory factors such as NO, TNF-α and IL. Moreover, the anti-inflammatory activities of pure borneol have been reported in several previous studies. For example, borneol could obviously loosen the inflammation in LPS-induced acute lung injury [6], alleviate mechanical hyperalgesia in models of chronic inflammatory and neuropathic pain in mice [30], and reverse oxygen-glucose deprivation followed by reperfusion-induced neuronal injury via anti-oxidation and anti-inflammation [3]. However, there is no report about the anti-inflammatory effect of iso-borneol compounds. Therefore, borneol would be the main bioactive compound in both NB and SB. The different amounts of borneol in the main chemical composition of NB (98.96%) and SB (61.39%) would contribute to the different bioactivity, as demonstrated in the LPS-induced inflammation cell model and fever rat model later.

Fever, the abnormal elevation of central body temperature, is perhaps the most common and best-known manifestation of an inflammation disease. A number of diseases induce fever either by raising the heat production of the body (hyperthyroidism) or by reducing heat loss (pheochromocytoma), but in the great majority of instances, fever is caused by an alteration in the central nervous system’s regulation of body temperature. Heat-clearing is a traditional effect of borneol, as a resuscitation-inducing aromatic medicine. Thus, we demonstrated the endotoxic fever relief effect of both NB and SB, in lowering elevated body temperature caused by intravenous injection of LPS. During 4 h post-injection of LPS, the maximum difference (ΔT) between fever body temperature and normal basal body temperature in the model, NB and SB groups was 2.12, 1.05 and 1.26 °C, respectively. This result indicated that, although both NB and SB could significantly reduce the fever temperature induced by LPS, NB would exhibit higher efficacy to improve the inflammation-involved fever. Furthermore, the different metabonomic profiles between NB and SB also support their unequal anti-fever activity. Our findings suggest that the impact metabolic pathways were the glycerophospholipid, linoleic acid, and alpha-linoleic acid metabolisms in LPS-induced fever rats. The glycerophospholipid (PC, PE, etc.) are key components of the lipid bilayer of cells, as well as being involved in metabolism and signaling. Previous studies have shown that lipids were the main different metabolites in the early stage, and subsequently influence energy metabolism and oxidative stress with LPS-treatment [31]. According to Edana Cassol et al. [32], lipid metabolism, including bile acids, sulfated steroids, and polyunsaturated fatty acids, were ligands of nuclear receptors that regulate inflammation, which correlated with markers of inflammation (interferon-α and interleukin-6). It has been shown that high density lipoprotein (HDL), very low-density lipoprotein (VLDL) or the lipid-free protein fraction of Lcat(-/-) mice, have a reduced capacity to reduce the TNF-α response in LPS-induced animals [33]. Moreover, the results also illustrated that there is still a great difference between NB and SB in alleviating endotoxic fever, by means of lipid metabolic pathways.

According to Figure 6, the difference on the metabolic profiles between the model group and SB treated group was non-significant, showing few blue variables in comparison results of the model group vs SB treatment group. It indicated that SB could scarcely regulate the abnormal metabolic pathways in the LPS-induced model group. This result was in accordance with the analysis result in Figure 5, in which the red score plots, representing SB group samples, was tightly close to blue score plots of model samples. On the contrary, there was a significant difference between NB and the model group in score plot results of PCA, PLS-DA and OPLS-DA analysis. Furthermore, we supplied the volcano plot comparison results between the NB treatment and SB treatment group in Figure 6, showing that there was a significant difference via amounts of blue variables. Interestingly, NB and SB exhibited similar anti-inflammatory factor results in RAW 264.7 cells and fevered rats, while there were significantly different metabolomic profiles. It would result in the fact that both physiological factors or body temperature regulation belong to the macroscopic manifestation of drugs on model animals. These indexes would suffer from the complex influences. However, the change of endogenic metabolites was more sensitive in reflecting the difference between diverse agents. Therefore, we employed the metabonomics approach to evaluate whether NB and SB possess equal bioactive mechanisms in anti-inflammatory action. Thus, it provided clear evidence that remarkable differences still existed between NB and SB in regulating the metabolites in fever rats.

A multicompartment, detailed targeted lipid-omics approach indicated that LPS can lead to disorders of fatty acid metabolism [34]. Previous reports have demonstrated that NO decreases the synthesis of fatty acids in isolated cultured rat hepatocytes, and, in a nitric oxide deficient hypertensive animal model, the protective effect of borneol on liver mainly changes the metabolic program of liver by increasing the structural modification of protein and triglyceride, as well as the quantitative change of protein, lipid and glycogen [35]. According to the previous study [36], LPS could result in a total of 27 disturbed metabolic pathways in the inflammation rat model. These metabolic pathways involved glutathione, cysteine, methionime, glycerophospholipid, d-glutamate, linoleic acid, alpha-linolenic acid, glycerolipid metabolism, valine, leucine, and isoleucine biosynthesis, arachidonic acid, glycine, serine, and threonine, pentose phosphate pathway, arginine and proline metabolism, and citrate cycle. Results on the abnormal metabolic pathways in this manuscript showed the involved glycerophospholipid, linoleic acid, and alpha-linolenic acid metabolism in Figure 8. Thus, these results were in accordance with the previous study. Also, 12 abnormal biomarkers, including fatty acids, amides, and organic acids were identified in the plasma of fever rats induced by LPS in present study. As shown in Figure 7, compared to the control group, the values of 12 biomarkers in the model group exhibited remarkable changes, namely a dramatic rise or decline. It suggested that the fever rats induced by intravenous injections of LPS had different metabolism profiles. However, both NB and SB could regulate the abnormal contents of these biomarkers at different degrees in comparison to the model group. For example, the level of fatty acid (14:0(10 Me, 13 Me)) was significant increased. Natural borneol can regulate the metabolism of fatty acids, but synthetic borneol has no such effect. Stearoylcarnitine is found in significantly greater amounts of patients with carnitine palmitoyltransferase (CPT) II deficiency, a rare disorder of lipid metabolism, in which the accumulation of long-chain acylcarnitines is a diagnostic marker [37].

## 4. Materials and Methods

### 4.1. Chemicals and Reagents

SB and NB samples were obtained from the National Institutes for Food and Drug Control (Beijing, China). LPS (batch No. 100M4095) was obtained from Sigma Chemical Co. (St. Louis, MO, USA). Mouse RAW 264.7 macrophage cell line was purchased from Shanghai Institute of Shanghai, China). DMEM high glucose medium (containing sodium acrylate), with the batch number of NZM1299 was purchased from Fisher Biochemical products Co., Ltd. (Beijing, China). Trypsin (0.25%) with the batch number of J140046 was purchased from HyClone laboratories. Fetal bovine serum (FBS) with the batch number of 120130 was bought from Caoyuan Lvye biological engineering materials Co., Ltd. (Hohhot, China). Penicillin streptomycin sulfate (double antibody) with the batch number of 07G10A16 was purchased from Wuhan Boshide Biological Engineering Co., Ltd. (Wuhan, China). MTT kit (No. C0009) and Griess kit (No. S0021) was purchased from Beyotime Institute of biotechnology. TNF-α Elisa kit (No. 228260752) and IL-6 Elisa kit (No. 220660823) were purchased from Hangzhou Lianke biotechnology Limited by Share Ltd. (Hangzhou, China). Methanol and acetonitrile with high-performance liquid chromatography (HPLC)-grade were purchased from Thermo Fisher Scientific Inc. (Waltham, MA, USA).

### 4.2. Chemical Profile by GC-MS Analysis

First, 50 mg of both NB and SB were extracted via ultrasonic extraction with 10 mL ethyl acetate for 20 min, respectively. The samples for GC-MS detection were obtained by centrifugation with 12000 rpm for 10 min and filtration through a 0.22 μm membrane filter. The relative contents of each chemical component in samples were calculated by area normalization method. GC-MS analysis was performed by HP-6890/5973 GC-MS (Agilent, Palo Alto, CA, USA) with a DB-WAX column (0.5 µm film thickness, 30 m lengths, 0.32 mm i.d.). The oven temperature was increased from 70 °C to 220 °C with a rate of 8 °C/min and then held isothermal for 10 min, with a total run time of 30 min. The temperature of the FID detector is 260 °C. The helium was used as carrier gas with a flow rate of 24.2 mL/min. The split-flow rate was 17.5 mL/min, and the split ratio was 10:1. The sample injection volume was set at 1 µL.

The chemical components in either NB or SB were identified from their GC retention indices (RI) obtained with reference, and comparison of their mass spectra and fragmentation patterns by computer matching with the Nist 8.0 Mass Spectral Database for GC-MS. The relative amounts of individual components were calculated based on GC peak area (FID response) without using a correction factor.

### 4.3. In Vitro Anti-Inflammation Effects on LPS-Induced RAW 264.7 Cells

The murine macrophage (RAW 264.7) cell line was cultured in DMEM high glucose medium supplemented with 10% (*v*/*v*) FBS, 100 U/mL penicillin and 100 μg/mL streptomycin at 37 °C in an atmosphere of 5% CO_2_. The RAW 264.7 cells were seeded in 96-well plates with 1 × 10^4^ /well overnight and then treated with LPS (1.0 μg/mL) for 30 min, followed by incubation with either NB or SB (50 mg/mL) for an additional 4 h or 24 h. 

The NO production in the cell-culture supernatant was determined by Griess reagent. Briefly, the culture media was collected and centrifuged at 10,000 rpm, 4 °C for 10 min. Then, 50 µL of Griess reagent was added into 50 µL of culture supernatant, following treatment according to the manufacturer’s instructions. Absorbance of each well was measured at 540 nm using ELISA micro-plate reader. Drew the standard curve with NO content as abscissa, absorbance OD as ordinate. According to the absorbance of the samples’ OD, the concentration of samples can be checked on the standard curve (μM). Similarly, the concentrations of pro-inflammatory cytokines including TNF-α and IL-6 in the cell-culture supernatant were determined by ELISA assay according to the manufacturer’s instructions. The absorbance of each well was determined at a maximum absorption wavelength of 450 nm and reference wavelength of 570 nm with dual wavelength detection by using a micro-plate reader. The sample concentration was calculated according to the standard curve.

### 4.4. Animal Handling for Endotoxic Fever Rat Model

Healthy SPF-grade adult male Sprague-Dawley rats (180 ± 20 g) were supplied by the Animal Breeding Center of DaShuo Biotechnology Co., Ltd. (Chengdu, China). All rats were maintained on an alternating 12 h light/dark cycle, at a temperature of 22–25 °C and humidity of 55–60%. The rats were fed with a standard diet and given free access to water. Experimental procedures were strictly in accordance with the Guide for the Care and Use of Laboratory Animals and were approved by the Animal Ethics Committee of Chengdu University of Traditional Chinese Medicine (TCM Pharmacology P3 laboratory, No. TCM2032043).

After acclimatization for 7 days, rats were randomly divided into four groups, including normal control group (control), fever group (model), SB group (250 mg/kg) and NB group (250 mg/kg). Rats in the model, NB and SB groups had fever induced by intravenous injection of LPS (5 mg/kg). Control group rats were gavaged with only normal saline, but without LPS treatment. Both SB and NB was freshly suspended in 0.5% CMC-Na, respectively. Rats in SB and NB groups were orally administered with 250 mg/kg of SB and NB at 0.5 h of LPS post-injection, respectively. After 3.5 h post-administration, the rats were anesthetized intraperitoneally with 1% pentobarbital sodium. Blood samples were collected from abdominal aorta. Serum samples were obtained by centrifugation isolation at 3500 rpm for 10 min and immediately stored at −80 °C for further use.

### 4.5. Serum Pretreatment

Serum samples were prepared for LC/MS analysis by protein precipitation with acetonitrile. Specially, 150 μL of cold acetonitrile was added into 50 μL of serum sample, vortexed for 60 seconds, and centrifuged at 13,500 rpm for 15 min at 4 °C. Then, the supernatant was collected for LC/MS analysis, and 3 μL of samples was injected for UPLC analysis.

### 4.6. UPLC-Q-TOF/MS Condition

Metabolomics analysis was performed with a 1260 Series Rapid Resolution Liquid Chromatography system (Agilent, Palo Alto, CA, USA) coupled to a Micro-TOF-Q II (Bruker, Karlsruhe,, German), equipped with an electrospray ionization source operating in positive ion mode. The serum sample analyses were performed with a Zorbax Eclipse Plus C8 Rapid resolution HT column (3.0 mm × 100 mm, 1.8 μm, Agilent). The gradient mobile phase was a mixture of 0.1% formic acid in water (A) and acetonitrile (B). The flow rate of 0.5 mL/min at 30 °C was used in linear gradients as follows: 95–50% A (0–3 min), 50–5% A (3–25 min), 5% A (25–35 min), 5–95% A (35–35.5 min), 95% A (35.5–40 min). The following MS parameters were employed: full scan range, 50 to 1200 *m*/*z*; drying gas flow, 6 L/min; source drying gas temperature: 250 °C; nebulizer pressure: 1.0 bar; capillary voltage: 5 kV. The MS/MS analysis was conducted with the normalized collision energy of 35 kV. 

A quality control sample was prepared by mixing 100 μL of each serum sample. The precision, repeatability of sample preparation, and system stability were validated before the experimental sample analysis.

### 4.7. Identification of Potential Biomarkers

The raw MS data were exported to MZmine2.9. The PCA, PLS-DA and OPLS-DA are frequently adopted for multivariate analysis. For biomarker identifications, the biochemical information of endogenous components was obtained and compared with databases like http://www.genome.jp/kegg/, http://www.hmdb.ca/, http://metlin.scripps.edu/, http://www.lipidmaps.org/, http://pubchem.ncbi.nlm.nih.gov/, http://www.chemspider.com/.

### 4.8. Statistical Analysis

All results were presented as the mean ± SD. Study data were analyzed using one-way analysis of variance (ANOVA) for significance comparison. Values of *p* < 0.05 were considered statistically significant.

## 5. Conclusions

In summary, several volatile components were identified in NB and SB by GC-MS. The main constituents of SB are iso-borneol and borneol, while that in NB is mainly borneol, and indicated the different chemical profiles between them. Although both NB and SB exhibited significant anti-inflammatory activity in vitro and anti-endotoxic fever effects in vivo, their efficacy in reducing the inflammation factors and body temperature are unequal. A metabolomics method has been established to investigate the potential mechanism to relieve LPS-induced fever in rats. Twelve potential biomarkers in serum were identified based on UPLC-Q-TOF/MS technology. The protective effect of NB and SB for inflammation has been reliably confirmed with the lipid metabolism. Therefore, the present study suggests that both NB and SB possess anti-inflammatory effects, in accordance with its traditional medicinal application. However, due to their different efficacies, the complete substitution of NB with SB still needs to be deliberated.

## Figures and Tables

**Table 1:** Characterization of components in synthetic borneol (SB) and natural borneol (NB) by gas chromatography-mass spectrometer (GC-MS).

Peak No.	R.T. (min)	Identity	Content% (SB)	Content% (NB)
1	2.16	Camphene	0.0016	0.0071
2	3.37	Cineole	-	0.0447
3	4.46	Fenchone	0.0038	-
4	6.04	Camphor	0.325	0.8095
5	6.73	Iso-butylene	0.0015	-
6	6.83	α-fenchol	0.4557	0.0116
7	6.93	β-fenchyl alcohol	0.3749	-
8	7.15	fenchyl acetate	-	0.0399
9	7.16	exo-methyl-camphenilol	0.0482	-
10	7.20	3-methyl-camphenilol	0.0151	0.001
11	7.49	m-menthene	0.0128	-
12	8.13	iso-borneol	35.77	0.0918
13	8.49	Borneol	61.39	98.96

**Table 2:** Identification results of the main potential biomarker changes.

Peak No.	R.T. (min)	Formula	Mass (*m*/*z*)	Biomarkers
1	6.04	C_16_H_35_NO_2_	274.2668	Sphinganine
2	1.14	C_5_H_11_N	85.0891	Piperidine
3	17.42	C_16_H_32_O_2_	256.2402	Fatty acid(14:0(10Me, 13Me))
4	9.64	C_26_H_52_NO_7_P	521.3554	PC(0:1/18:1(9E))
5	15.90	C_20_H_34_O_2_	306.2559	3β,5β-androstanediol
6	1.12	C_7_H_13_NO_3_	159.0895	5-acetamidopentanoate
7	18.13	C_18_H_34_O_2_	282.2559	*cis*-2-octadecenoic acid
8	21.44	C_40_H_79_O_7_P	702.5563	PA(P-18:0/19:0)
9	8.78	C_23_H_45_NO_4_	399.3349	Palmitoylcarnitine
10	23.82	C_44_H_84_NO_8_P	785.6007	PC(18:1(11Z)/18:1(9Z))
11	18.12	C_42_H_80_NO_8_P	757.5622	PE(22:2(13Z, 16Z)/15:0)
12	10.33	C_25_H_49_NO_4_	427.3662	DL-stearoylcanitine
